# Characterization of Novel Microsatellite Loci for *Primula poissonii* (Primulaceae) Using High-Throughput Sequencing Technology

**DOI:** 10.3390/molecules21050536

**Published:** 2016-05-09

**Authors:** Yun-Jiao Liu, Cai-Yun Zhang, Gang Hao, Xue-Jun Ge, Hai-Fei Yan

**Affiliations:** 1 Key Laboratory of Plant Resources Conservation and Sustainable Utilization, South China Botanical Garden, Chinese Academy of Sciences, Guangzhou 510650, China; lyj1350802@126.com (Y.-J.L.); xjge@scbg.ac.cn (X.-J.G.); 2 College of Life Sciences, South China Agricultural University, Guangzhou 510642, China; cyzhang@scau.edu.cn (C.-Y.Z.); haogang@scau.edu.cn (G.H.)

**Keywords:** *Primula poissonii*, 454 pyrosequencing, SSR, genetic diversity, speciation events, transferability

## Abstract

*Primula poissonii* (Primulaceae) is a perennial herb, widely distributed in the Hengduan Mountain region of Southwest China. In this study, Roche 454 pyrosequencing was used to isolate microsatellite markers. A total of 4528 unique sequences were identified from 68,070 unique reads. Of these, eighty-seven microsatellite loci were screened for utility using two criteria: successful PCR amplification and variation of these loci within three wild *P. poissonii* populations. Twenty loci were successfully amplified and exhibited polymorphic alleles. The number of observed alleles ranged from 1 to 9 with an average of 3.5. The observed and expected heterozygosities ranged from 0.087 to 1.000 and from 0.124 to 0.828, respectively. Among these SSR loci, only the P69 locus could not be cross-amplified successfully in two closely related species *P. wilsonii* and *P. anisodora*. The microsatellite loci developed in this study will be useful for studying genetic diversity and speciation events between *P. poissonii* and closely related *Primula* species.

## 1. Introduction

The Hengduan Mountain region in southwestern China is considered as one of the most important biodiversity hotspots in the world [1]. The total number of spermatophytes in the region totals 8590, with 2783 (32.4%) being endemic to the region [2]. *Primula* L. (Primulaceae) is comprised of more than 500 species worldwide, with 300 distributed in China [3]. With over 100 *Primula* species narrowly distributed within the Hengduan Mountains, this region has received considerable attention from biologists [4,5,6,7].

*Primula poissonii* Franch. is a perennial herb, always found growing in wet areas or inundated meadows, at an average elevation of 2500–3100 m.a.s.l. [3]. It is an endemic species restricted to the Hengduan Mountains [3]. The complex topography of the Hengduan Mountains and climatic oscillation during the Quaternary period might have deeply affected the population structure and genetic diversity of *P. poissonii* [7]. Studies of *P. poissonii* with polymorphic markers (e.g., microsatellites) can serve as a model to help illuminate the historical changes of the Hengduan Mountains, just as *P. mistassinica* has for the boreal regions [8] and *P. halleri* for the alpine areas [9].

Microsatellite markers have been considered to be effective in assessing the genetic diversity and structure of plant populations [10,11,12,13]. A number of EST-SSR primers for *P. poissonii* were recommended by Zhang *et al.* [14]. EST-SSRs are particularly useful in exploring the genome for possible signatures of natural selection, however, they often exhibit non-neutrality [15]. Hence, high polymorphic and neutral genomic SSR markers would be better resources to assess the genetic diversity and differentiation of natural populations of *P. poissonii*. Recently, next-generation sequencing technology has been widely used in many areas, including the development of novel microsatellite markers [8,10,11]. In this study, we developed a set of variable microsatellite markers using the Roche 454 pyrosequencing platform. These markers will likely to be important tools for investigating genetic diversity, population structure, and speciation history of *P. poissonii* and closely related taxa.

## 2. Results and Discussion

Identified unique reads totaled 68,070, ranging from 300 to 600 base pairs. A total of 7615 unique reads contained at least one microsatellite motif, of which 4528 unique sequences were identified with sufficiently large flanking regions suitable for primer design (the sequences in these reads are available upon request). The microsatellite sequencing efficiency using Roche 454 high-throughput sequencing technology in *P. poissonii* (59.5%) was higher than that in *Primula mistassinica* (31.2%) [8] or *Primula veris* (22.2%) [16].

Eighty-seven microsatellite loci were randomly selected to test their performance in PCR amplification to assess polymorphism in three populations of wild *P. poissonii*. In these loci, perfect dinucleotide repeats were the most abundant (77.0%), with (AC)_n_/(TG)_n_/(CA)_n_/(GT)_n_ being the most common motifs (50.7%).

All primer pairs were tested in nine *P. poissonii* individuals by using PCR amplification. Successful amplification was checked by running PCR product out on 2% agarose gels. PCR product showing a single, clear band on agarose gels was sequenced to verify the existence of corresponding repeat motifs. Twenty-five primer pairs which could generate clear PCR product of appropriate size (100–300 bp) and with repeat motifs, were resynthesized with fluorescent FAM or HEX labels. Subsequent amplification trials were conducted in all 77 individuals from three wild populations.

Five loci could not be clearly characterized and were therefore discarded in the following analyses. The characters of the remaining 20 polymorphic loci are listed in Table 1. Genetic diversity parameters for three wild *P. poissonii* populations are shown in Table 2. The number of alleles (*A*) varied from 1 to 9, with an average of 3.5. Among polymorphic loci, the observed heterozygosity (*H*_O_) and expected heterozygosity (*H*_E_) ranged from 0.087 to 1.000, 0.124 to 0.828, respectively. The inbreeding coefficient (*F*is) ranged from −1.000 to 0.710. Some loci deviated significantly from Hardy-Weinberg equilibrium (HWE) in several populations (Table 2), mainly result of heterozygote deficiency, such as locus P16 in the Lijiang population (LJ). The presence of null alleles, Wahlund effect, and small population size might cause an excess of homozygotes [17]. In this study, only P4, which deviated from Hardy-Weinberg equilibrium in population LGH, can be attributed to the presence of null alleles detected by MICRO-CHECKER (Table 2). The limited sampling ranges of each population is likely the leading cause of the observed excess of homozygotes.

The *P. poissonii* species complex is part of a taxonomically challenging group, which consists of *P. anisodora* Balf. f. et Forr., *P. wilsonii* Dunn and *P. poissonii*. This complex is a good system to test species boundary, genetic introgression and speciation events in closely related taxa, especially those that may have experienced rapid evolutionary radiation. Yan *et al.* [18] confirmed that the *P. poissonii* complex is resolved as monophyletic with high support by DNA barcodes, but the exact relationships amongst these species is still unclear. Therefore, other reliable molecular markers (such as microsatellites) will facilitate further speciation studies of the complex. We conducted cross-species amplification tests in *P. wilsonii* and *P. anisodora* using the successful 20 microsatellite markers. Nineteen of the 20 microsatellite loci were amplified successfully, only the locus P69 failed (Table 2). This result demonstrates that the microsatellite primers developed in this study can be widely used in *P. poissonii* and its relatives (*P. wilsonii* and *P. anisodora*).

## 3. Experimental Section

### 3.1. Plant Materials and Genomic DNA Extraction

Seventy-seven individuals of *P. poissonii* were collected from three wild populations in the Hengduan Mountain region, in Southwest China. LJ (100°10′ E; 27°00′ N; voucher specimen No.: Y2013051) and LGH (100°44′ E; 27°42′ N; voucher specimen No.: Y2013075) populations both located in Yunnan Province, while population YY (101°42′ E; 27°31′ N; voucher specimen No.: Y2013087) is from Yanyuan, Sichuan Province. Leaf materials of *P. anisodora* (voucher specimen No.: Y2013062) and *P. wilsonii* (voucher specimen No.: Y2013015) were collected from Zhongdian, Yunnan Province and Ailao Mountain, Yunnan Province, respectively. Voucher specimens were deposited at the herbarium of the South China Botanical Garden (IBSC). Total genomic DNA was extracted following a modified version of the cetyltrimethyl ammonium bromide (CTAB) protocol of Doyle and Doyle [19].

### 3.2. SSR-Enriched Library Construction and 454 Sequencing

A shotgun library was prepared by shearing 1 μg of genomic DNA using the DNA Library Preparation Kit (Roche Applied Science, Indianapolis, IN, USA) following the GS FLX+ library preparation protocol. The shotgun library was further enriched by the biotin-capture method [20,21]. Eight 5’-biotinylated probes, which were recommended by Toth *et al.* [20] and Zane *et al.* [21], were used in this study, namely, (AG)_10_, (AC)_10_, (AAC)_8_, (ACG)_8_, (AAG)_8_, (AGG)_8_, (ACAT)_6_, and (ATCT)_6_. The microsatellite-enriched library was sequenced on a Roche 454 GS FLX platform.

### 3.3. Detection of SSR Markers and Primer Design

MISA software [22] was used to identify unique reads containing microsatellite repeats. The search was performed for six for di-, five for tri- and tetranucleotides repeats, respectively. And the parameter of a minimum product size was set to 100 bp. Primer3 software [23] was applied to design primer pairs. The minimum primer annealing temperature was set to 60 °C, primer size was between 18–22 bp with an optimal size of 20 bp, and other setting were performed with default values.

### 3.4. Amplification of SSR and Genotyping

PCR reactions were done in a PTC-200 Thermal Cycler (MJ Research, Watertown, MA, USA). Conditions of PCR amplification were as follows: an initial denaturation at 95 °C for 5 min; followed by 30 cycles at 94 °C for 30 s, locus-specific annealing temperature (Table 1) for 45 s, and 72 °C for 50 s; with a final extension at 72 °C for 7 min. All primer pairs were initially tested for successful PCR amplification in nine *P. poissonii* individuals on 2% agarose gels. PCR products which were single, clear bands on agarose gels were sequenced to check the existence of repeat motifs. Primer pairs in the first trial which could generate clear PCR product with repeat motifs were then resynthesized with a fluorescent dye (FAM or HEX). Subsequent analyses were conducted with the polymorphic markers for all 77 individuals of three wild *P. poissonii* populations. Thereafter, 1 μL of the PCR product containing the fluorescent dye-labeled fragments was added to 8.8 μL of formamide and 0.2 μL of GeneScan 500 ROXSize Standard (Applied Biosystems, Life Technologies, Woolston, Cheshire, UK) and subsequently run on an ABI PRISM 3130 Genetic Analyzer (Applied Biosystems).

### 3.5. Data Analysis

Genotypes were called using Gelguest software (version 3.2.1; SequentiX-Digital DNA processing, Klein Raden, Germany). Genetic diversity parameters, allelic richness (*A*), observed and unbiased expected heterozygosity (*H*_O_, *H*_E_), and the inbreeding coefficient, were estimated using GenAlEx 6.5 [24,25]. Deviations from Hardy-Weinberg equilibrium (HWE) at each locus were tested using GENEPOP 4.0.7 [26]. Null alleles were detected by MICRO-CHECKER [27].

### 3.6. Validity of the Transferability of SSR Markers

Cross-species amplification tests were performed in *P. wilsonii* and *P. anisodora*, which are both morphologically close to *P. poissonii*. Primer transferability was considered successful when one clear distinct band of PCR product in the expected size range was apparent on 2% agarose.

## 4. Conclusions

In this study, we characterized twenty microsatellite loci for *P. poissonii*. These markers displayed high levels of polymorphism in three wild populations. Nineteen of these loci could be successfully amplified in the two other closely related *Primula* taxa (*P. anisodora* and *P. wilsonii*). These microsatellite markers will provide insight on population genetic structure and be useful for phylogeographic analyses. They will also help to detect the hybridization and/or genetic introgression between these three taxa in the *P. poissonii* complex.

## Figures and Tables

**Table 1:** Characteristics of 20 microsatellite loci developed in *Primula poissonii*.

Locus	Primer Sequences (5′-3′)	Repeat Motif	Fragment Size Range (bp)	Tm (°C)	GenBank Accession No.
PP4	F:TTGAGAGGGGTGTTTTGGTC	(AG)12	226–246	60	KU744608
	R:CACCTCTCTTCCCCTCTTCC				
PP10	F:TCTCGTCTTTTCCGCAAACT	(AT)15	238–256	60	KU744609
	R:TCATCATCATCACACGCAAA				
PP13	F:AATCTTCTAACACGCATTCACTC	(AT)13	165–163	57.5	KU744610
	R:GGGGTAGATTGAATGTTTCCG				
PP14	F:CCAAACCAAACACATTGGAA	(AT)11	258–271	60	KU744611
	R:GGCATAGGTTGTTCCTGCAT				
PP16	F:TCCAGTGACCACTCTGCTTG	(CTT)14	280–286	60	KU744612
	R:ATGGTGCTTAATTCGTTGCC				
PP17	F:GCCTCAATCTGCCACTCAAT	(CA)19	168–190	55.5	KU744613
	R:AAGCTCCTGGTTGAAAGGGT				
PP27	F:TCATGAATTCGGACTGGAGA	(AG)11	136–150	55.5	KU744614
	R:AGCACTTCATTTGGTGAGCC				
PP31	F:TCGGTCTATGATTTGGAATGG	(TG)13	146–150	59	KU744615
	R:TCAACATGCCTCTACAAGCG				
PP37	F:GGAAAATCAATCTTGGGGGT	(AAC)14	231–256	60	KU744616
	R:TTGTACCCGTCATCGGATTT				
PP38	F:TTATCACTGCCATTTGGCTAC	(TTC)14	188–223	60	KU744617
	R:CATGGCACTTTGTCATTTAGGA				
PP48	F:ACTAGCAACAACCCAAACGG	(CA)16	288–300	60	KU744618
	R:ATAGGGACGTTGATCGCTTG				
PP49	F:AGGGAACTACACGTTTGGCA	(TG)16	201–211	60	KU744619
	R:AACACCTGCCACATCACTTG				
PP50	F:TCACAAGCATTTTATCGGCA	(CA)16	198–202	60	KU744620
	R:AAATGGGTGAGTTTGCGAAG				
PP52	F:AAATAGGTCCGCATACGTGC	(AC)15	300–310	60	KU744621
	R:TTATGAAGTATGTTGTGTATGAAGCA				
PP62	F:TTCATCTTGATTAATTGGGGG	(TA)14	231–252	60	KU744622
	R:TCACCATTTTCACTAGACGCA				
PP69	F:GTGAATGGGTAGCGGACAAT	(AT)11	336–354	60.7	KU744623
	R:GGTGCCGAAGGTGCTTAG				
PP72	F:ATCCAAGATGAGCAACTCCG	(TCT)7	184–209	60	KU744624
	R:CCATTCATCTCAACAATAGATTTCC				
PP75	F:CGAGCGTGTCCTGGATATTT	(AT)10	210–216	60	KU744625
	R:ATTTCTCCGGATTCGTCACA				
PP81	F:CCTCCCCCTTCTTCATTCTC	(AG)11	140–152	60.7	KU744626
	R:ACCTCTCCTCCGTCTTCCTC				
PP84	F:TGCTTAGTGCTAAACAACTCATTG	(TGT)8	117–120	60	KU744627
	R:CCGGAATTCTCTCCTCCTTC				

**Table 2:** Population genetic parameters estimated (per nSSR locus) in three populations and two species of *Primula* section *Proliferae*.

*Primula poissonii*															
Locus	LJ (*n* = 25)				LGH (*n* = 23)				YY (*n* = 29)				Total A	*Primula wilsonii*	*Primula anidosora*
	A	Ho	He	Fis	A	Ho	He	Fis	A	Ho	He	Fis			
P4	9	0.960	0.782	−0.208	7	0.261	0.453 ^a,b^	0.442	5	0.483	0.646 ^b^	0.269	13	+	+
P10	6	0.240	0.566 ^b^	0.590	4	0.261	0.601 ^b^	0.581	3	0.759	0.514	−0.463	9	+	+
P13	1	NA	NA	NA	3	0.217	0.419	0.498	3	0.241	0.395	0.404	3	+	+
P14	8	0.960	0.762	−0.241	7	0.957	0.739	−0.274	5	0.931	0.644	−0.432	10	+	+
P16	2	0.200	0.180 ^a^	−0.091	3	0.696	0.480	−0.431	2	0.607	0.423	−0.421	4	+	+
P17	7	0.720	0.766	0.081	5	0.478	0.520 ^a^	0.028	5	0.207	0.193 ^a^	−0.057	8	+	+
P27	9	0.560	0.798 ^b^	0.317	5	0.409	0.574	0.309	2	0.172	0.158 ^a^	−0.077	15	+	+
P31	4	0.160	0.403 ^b^	0.616	3	0.136	0.245	0.462	3	0.862	0.609	−0.400	4	+	+
P37	7	0.640	0.743	0.159	3	0.130	0.124 ^a^	−0.031	3	0.517	0.595	0.147	8	+	+
P38	8	0.360	0.696 ^b^	0.498	2	0.696	0.454	−0.517	8	0.828	0.828	0.018	13	+	+
P48	2	0.680	0.449	−0.500	3	1.000	0.521	−0.917	3	0.138	0.461 ^b^	0.710	4	+	+
P49	3	0.619	0.441	−0.383	1	NA	NA	NA	3	0.143	0.135 ^a^	−0.044	4	+	+
P50	3	0.800	0.563	−0.404	2	0.087	0.227 ^b^	0.630	3	0.552	0.469	−0.159	3	+	+
P52	4	0.304	0.268 ^a^	−0.112	2	0.773	0.474	−0.615	2	0.552	0.400	−0.366	4	+	+
P62	3	0.320	0.274 ^a^	−0.146	3	0.652	0.464	−0.387	4	0.241	0.221 ^a^	−0.077	5	+	+
P69	5	1.000	0.574	−0.732	4	1.000	0.593	−0.676	3	1.000	0.624	−0.579	9	−	−
P72	4	0.160	0.151 ^a^	−0.038	3	0.609	0.549 ^a^	−0.086	8	0.241	0.565 ^b^	0.584	11	+	+
P75	3	0.880	0.534	−0.635	2	1.000	0.500	−1.000	3	1.000	0.618	−0.608	4	+	+
P81	3	0.720	0.569	−0.247	4	0.565	0.479 ^a^	−0.158	3	0.690	0.546 ^a^	−0.246	6	+	+
P84	2	0.600	0.420 ^a^	−0.412	1	NA	NA	NA	1	NA	NA	NA	2	+	+
Mean	4.650	0.544	0.497	−0.117	3.250	0.496	0.419	−0.135	3.600	0.508	0.452	−0.110	6.950		

+ = successful PCR amplification; – = unsuccessful PCR amplification; A = number of alleles per locus; He = expected heterozygosity; Ho = observed heterozygosity; Fis = fixation index frequency; ^a^: Significant deviation from Hardy–Weinberg equilibrium (*p* < 0.05); ^b^: Significant frequency of null alleles.

## Abbreviations

The following abbreviations are used in this manuscript:

PCR	Polymerase Chain Reaction
SSR	Simple Sequence Repeat
Mts	Mountains
EST-SSR	Expressed Sequence Tag Simple Sequence Repeat
HWE	Hardy-Weinberg Equilibrium
He	Expected Heterozygosity
Ho	Observed Heterozygosity
Fis	Fixation Index Frequency
